# EHD1 Functions in Endosomal Recycling and Confers Salt Tolerance

**DOI:** 10.1371/journal.pone.0054533

**Published:** 2013-01-14

**Authors:** Maya Bar, Meirav Leibman, Silvia Schuster, Hilla Pitzhadza, Adi Avni

**Affiliations:** Department of Molecular Biology and Ecology of Plants, Tel-Aviv University, Tel-Aviv, Israel; National Taiwan University, Taiwan

## Abstract

Endocytosis is a crucial process in all eukaryotic organisms including plants. We have previously shown that two Arabidopsis proteins, AtEHD1 and AtEHD2, are involved in endocytosis in plant systems. Knock-down of EHD1 was shown to have a delayed recycling phenotype in mammalians. There are many works in mammalian systems detailing the importance of the various domains in EHDs but, to date, the domains of plant EHD1 that are required for its activity have not been characterized. In this work we demonstrate that knock-down of EHD1 causes a delayed recycling phenotype and reduces Brefeldin A sensitivity in Arabidopsis seedlings. The EH domain of EHD1 was found to be crucial for the localization of EHD1 to endosomal structures. Mutant EHD1 lacking the EH domain did not localize to endosomal structures and showed a phenotype similar to that of EHD1 knock-down seedlings. Mutants lacking the coiled-coil domain, however, showed a phenotype similar to wild-type or EHD1 overexpression seedlings. Salinity stress is a major problem in current agriculture. Microarray data demonstrated that salinity stress enhances the expression of EHD1, and this was confirmed by semi quantitative RT-PCR. We demonstrate herein that transgenic plants over expressing EHD1 possess enhanced tolerance to salt stress, a property which also requires an intact EH domain.

## Introduction

Eukaryotic cells require endocytosis for uptake of extra-cellular substances and internalization of plasma membrane proteins for transport to endosomes [1]. Endocytosis regulates and is involved in many important processes, including several signaling pathways [2], [3], [4].

Plants require endocytosis for important processes including development [5] and defense against microorganisms [6], [7]. Studies conducted in plant systems have elucidated possible functionalities of plant endocytic compartments and the flow of endocytosed material throughout plant cells [7], [8], [9], [10], [11], [12], [13], [14].

Endocytosis depends on a large number of protein-protein interactions mediated by specific modules. One such module is the EH (Eps15 homology) domain first identified in Eps15 [15], [16]. The EH domain structure generally consists of two EF-hands and a helix-loop-helix structure that binds calcium (or a pseudo EF-hand), connected by an anti-parallel beta-sheet [17], [18], [19]. Many EH-containing proteins were identified in different species, among them EHD1-4 (EH domain containing proteins), Eps15 and Intersectin 1–2 [20], [21], [22], [23].

Four EHD orthologs are known in vertebrates [24] and two in plants [25]. All mammalian EHDs share a similar structure: An N-terminal domain with a nucleotide binding motif (P-loop), DxxG and NKxD, a central coiled coil region and a C-terminal EH domain containing an EF Ca^2+^ binding motif. C-terminal EH domain containing proteins are regulators of endocytic trafficking, and have been shown to associate with Rab protein effectors [24], [26]. Despite their high homology (70–80%) the mammalian EHDs differ in the transport steps which they regulate [20], [27], [28], [29].

Mammalian EHD1 was shown to regulate the recycling of many receptors [30], endocytosed via both clathrin [31] and non clathrin pathways [32], [33]. Based on the knowledge to date, EHD1 is involved primarily in recycling from the endocytic recycling compartment (ERC) to the plasma membrane. In addition, evidence suggests that EHD1 is involved not only in recycling to the plasma membrane, but also in transport of receptors from the early endosome to the ERC [26], [34], as well as in retrograde transport from endosomes to golgi [35]. EHD3, which shares the highest level of homology with EHD1 amongst the mammalian EHD proteins, is also involved in endosome to golgi transport and appears to be required for maintenance of golgi morphology and function [36].

We previously reported the isolation and characterization of two Arabidopsis EH domain containing proteins (AtEHD1 and AtEHD2; [25] Both proteins contain an EH domain with two EF calcium binding hands, a P-loop, with a predicted ATP/GTP binding site, a bipartite NLS and a coiled-coil domain, as well as a Dynamin-N motif. AtEHD1 was found to be involved in endocytosis in plant systems, and knock-down of AtEHD1 was found to delay internalization of endocytic cargo, perhaps indicating a delay in recycling as was reported for EHD1 knock-out mice [29].

Here we report that AtEHD1 localizes to RabA and RabD positive vesicles, functions in endocytic recycling in plant cells, and requires an intact EH domain to do so. We found that overexpression of EHD1 leads to increased salinity stress tolerance and decreased ROS accumulation during salinity stress, perhaps indicating a correlation between endocytic recycling and plant stress coping mechanisms.

## Results

### EHD1 is localized to RabA and RabD positive vesicles

**Figure 1:**
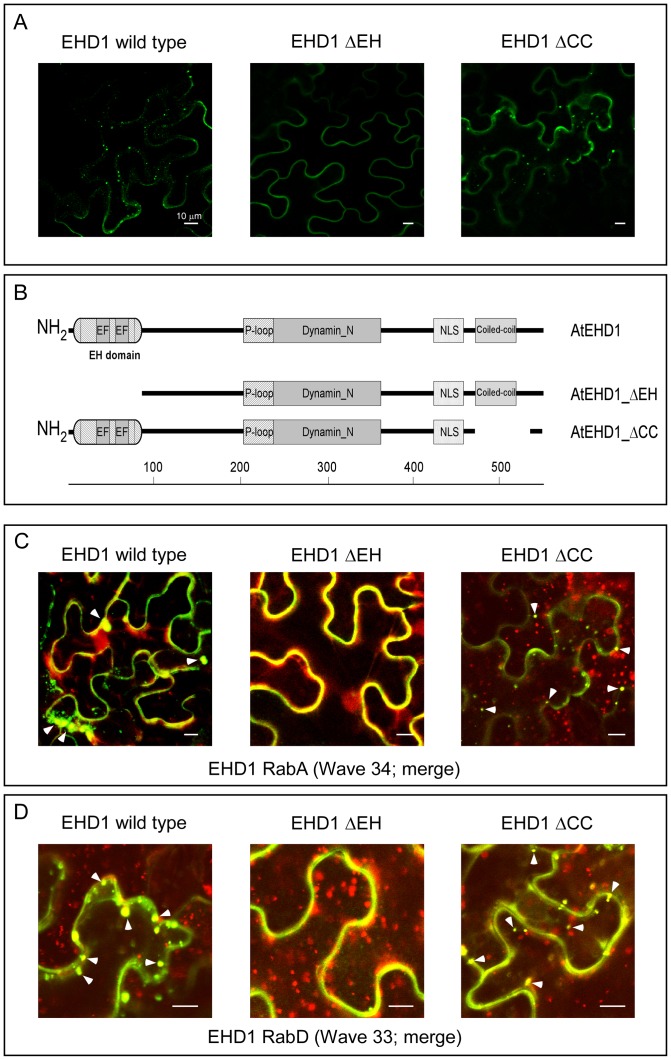
Localization of AtEHD1 and mutant forms and co-localization with a Wave marker. (A) Localization of wild-type and deletion mutant EHD1 proteins. Tobacco plants transiently expressing AtEHD1-GFP (left), AtEHD1-ΔEH-GFP (middle) and AtEHD1-ΔCC-GFP (right). Leaf sections were visualized under a laser-scanning confocal microscope. Scale bar  = 10 µm. (B) Schematic representation of AtEHD1 mutant forms. ΔCC  =  truncation mutant lacking coiled-coil domain (amino acids 466–481). ΔEH  =  truncation mutant lacking EH domain (amino acids 94–545). (C); (D) co-localization of wild-type and deletion mutant EHD1 proteins with RabA and RabD WAVE markers. Tobacco plants transiently expressing AtEHD1-GFP (left), AtEHD1-ΔEH-GFP (middle) and AtEHD1-ΔCC-GFP (right) and co-expressing Rab A1e-WAVE 34 (C) and RabD2b-WAVE33 (D). Leaf sections were visualized under a laser-scanning confocal microscope. Scale bar  = 10 µm. Arrowheads indicate co-localized pixels.

Overexpression of an EHD1-GFP fusion exhibits membranal and vesicular localization in tobacco and Arabidopsis cells [25]; Figure 1A). We have previously demonstrated that the vesicular structures containing EHD1 are endosomal and co-localize with the FYVE domain, particularly in the vicinity of the membrane. In order to obtain insight into EHD1 function, we searched for additional marker proteins which co-localize with EHD1. Following publication of the WAVE toolbox set of membrane protein fluorescent tags [37], we proceeded to examine the localization of WAVE lines which were reported to reside on endosomes with EHD1. We found that EHD1 co-localizes with Waves 33 and 34 (Figure 1C, D). Wave 34 is classified in plants as RabA1e, a homolog of mammalian Rab11. RabA1e was shown to localize to endosomes, possibly recycling endosomes in plant cells, and to have high BFA sensitivity [38], [39], [40], [41]. Further, we also found EHD1 to co-localize with Wave line 33, which belongs to the RabD family and was described to possess endosomal and golgi localization. While we have previously confirmed that EHD1 does not localize to golgi bodies *per se* [25], it would seem that the plant RabD proteins localize to both golgi and non-golgi endosomal compartments which are BFA sensitive [13], [42]. Indeed, the RabD proteins examined in our study appear to localize to additional vesicles which do not contain EHD1.

Further evident from Figure 1, is the fact that while an EHD1 mutant lacking the coiled-coil domain (amino acids 1–465 fused to amino acids 482–545 of EHD1; see Figure 1B) continues to reside on endosomal structures and co-localizes with RabA/RabD proteins (Figure 1C, D), though it possesses a reduced membrane presence, an EHD1 mutant lacking the EH domain (amino acids 94–545 of EHD1, figure 1B) is excluded from RabA/RabD containing vesicles (Figure 1C, D), and is almost exclusively membranal. The EH domain appears to be critical for the vesicular localization of EHD1.

### EHD1 is involved in recycling

**Figure 2:**
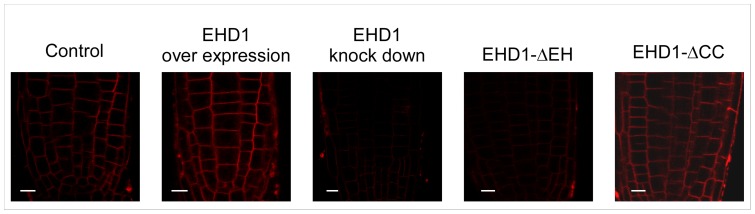
Internalization of Fm-4-64 in Arabidopsis seedling roots. 7–10 day old wild-type or transgenic seedlings (as indicated) were floated on a 5 µM FM-4-64 solution for 5 minutes and then washed. Root sections were visualized under a laser-scanning confocal microscope. Scale bar  = 10 µm.

**Figure 3:**
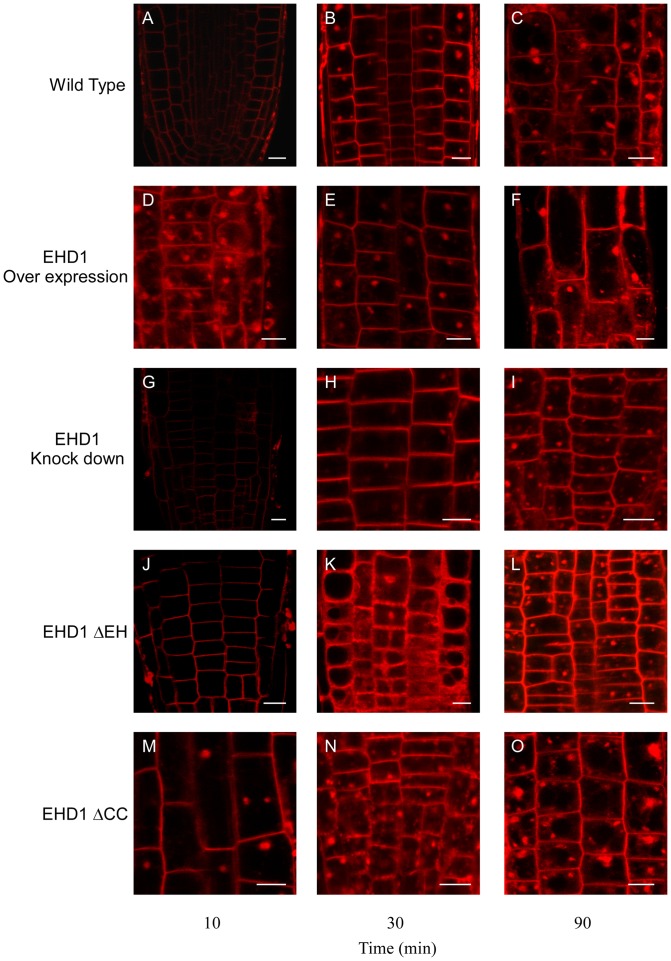
Effect of BFA treatment on Arabidopsis seedling roots. 7–10 day old transgenic seedlings were floated on a 50 µM BFA solution supplemented with 5 µM Fm-4-64 for different time points (as indicated) and then washed. Root sections were visualized under a laser-scanning confocal microscope. (A–C) wild type; (D–F) EHD1 overexpressing; (G–I) EHD1 knock-down; (J–L) EHD1-ΔEH overexpressing; (M–O) EHD1-ΔCC overexpressing. Scale bar  = 10 µm.

As discussed above, mammalian EHD1 is involved in endocytic recycling in several systems. We have previously shown that Arabidopsis plants knocked-down in EHD1 internalize Fm-464 in a delayed time frame as compared with wild-type plants [25]; Figure 2). Here we show further that a deletion in the coiled-coil domain does not possess delayed Fm-4-64 internalization (Figure 2), while a deletion in the EH domain behaves like a knock-down mutant and also possesses delayed Fm-4-64 internalization (Figure 2). Since EHD1 deficient transgenic mice were shown to have a delayed recycling phenotype [29], we suspected that perhaps delayed internalization of Fm-4-64 was indicative of a similar phenotype in plants. In order to further analyze recycling in Arabidopsis, we examined the effect of Brefeldin A (BFA) on transgenic Arabidopsis plants. A drug which impairs recycling will have a lower/lesser effect on cells in which recycling is diminished, and therefore BFA sensitivity is decreased. Figure 3 depicts the results of this analysis. Typically, the large endosomal aggregations seen after BFA treatment termed a “BFA body” will appear in wild-type Arabidopsis root cells after 30 minutes [43]; Figure 3). EHD1 knock-down plants did not generally form BFA bodies after 30 minutes of treatment (Figure 3H); Interestingly, plants overexpressing EHD1 exhibited BFA bodies in an accelerated time frame, after only 10 minutes of BFA treatment (Figure 3D; compare with wild-type cells in the same time point, Figure 3A), suggesting that overexpression of EHD1 may cause enhanced/accelerated recycling, leading to increased BFA sensitivity. EHD1 can be found in the BFA bodies following BFA treatment (Figure S1) confirming EHD1 to be BFA sensitive, as was also indicated for the RabA/RabD proteins with which it co-localizes [37], leading to the conclusion that it is indeed localized to BFA sensitive compartments. Interestingly, EHD1_ΔEH can be seen in the vacuole following BFA treatment, while EHD1_ΔCC localized to the BFA bodies (Figure S1). These experiments led us to the conclusion that Arabidopsis plants knocked-down in EHD1 are delayed in recycling while plants overexpressing EHD1 may possess enhanced recycling; we next examined the two deletion mutants. Figure 3J–I show that the EH domain deletion mutant behaves essentially like an EHD1 knock-down, possessing decreased BFA sensitivity, while the coiled-coil domain deletion mutant behaves essentially like EHD1 overexpression (Figure 3M–O), possessing increased BFA sensitivity. EHD2 knock-down seedlings behaved similarly to wild-type seedlings throughout the course of the experiment (Figure S2).

### Overexpression of EHD1 confers salt tolerance

**Figure 4:**
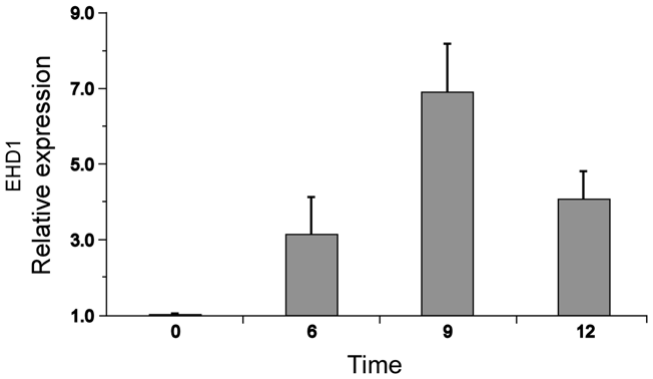
Relative expression of endogenous EHD1 in response to salt treatment. Arabidopsis seedlings were treated with 200 mM Nacl at time points as indicated. cDNA was prepared followed by semi quantitative RT-PCR reactions using specific primers to EHD1. RT-PCR products were separated on an agarose gel, stained with ethidium bromide and quantified using ImageJ software. Relative expression of EHD1 compared to untreated cells at time sero is presented. Each point represents the average ± SE of 3 different experiments.

**Figure 5:**
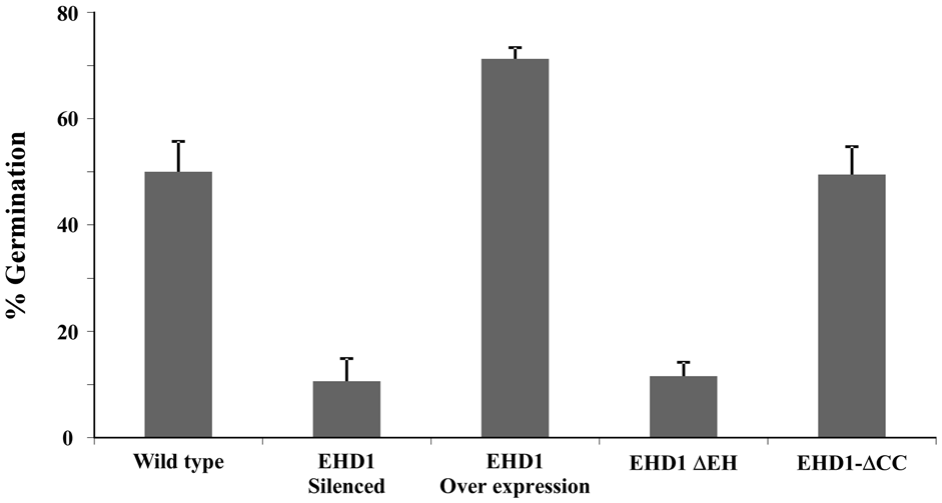
Effect of salt treatment on seed germination. Arabidopsis seeds were gereminated on 200 mM NaCl. Germination was normalized based on the germination values on media without NaCl. Values represent mean ± SE of 6 experiments.

Analyzing the expression pattern of EHD1 revealed that its expression increases following salt stress [44]. We confirmed this observation by semi-quantitative RT-PCR, determining that 9 hours following salinity treatment (200 mM NaCl for indicated time points, see Figure 4) EHD1 reaches a peak of 7 times the level of its basal expression. EHD2 has extremely low endogenous expression [25], often below the threshold of detection; this did not change throughout the course of this experiment. To further examine a possible connection between EHD1 function and salt tolerance we exposed EHD1 overexpressing and knock-down seedlings to salt stress. The expression of EHD1, ΔEH and ΔCC were monitored in the transgenic plants (Figure S3). As can be seen in Figure 5, EHD1 overexpressing seedlings possess increased salt tolerance, as is evident from their increased ability to germinate on NaCl containing media. Perhaps not surprisingly, seedlings knocked-down in EHD1 have increased NaCl sensitivity as compared with wild-type seedlings. Once again, the deletion in the EH domain behaves like an EHD1 knock down, while, in this specific case, the deletion in the coiled-coil domain did not confer increased germination on salt containing media, behaving instead like the wild type seeds. EHD2 knock-down seedlings behaved similarly to wild-type seedlings throughout the course of the experiment (Figure S2).

**Figure 6:**
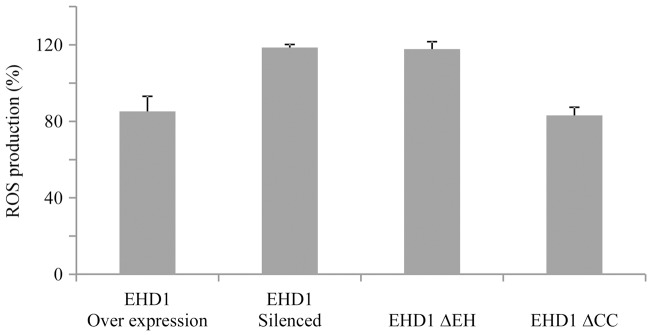
ROS production in Arabidopsis seedlings. 7–10 day old transgenic seedlings (as indicated) were floated on a 200 mM NaCl solution. ROS were quantified after 2 hours of treatment. Values are normalized against ROS production in wild type seedlings. Values represent mean ± SE of 3 experiments.

Salt sensitivity in Arabidopsis has been correlated with an increase in reactive oxygen species [45],[46]. We examined the production of ROS with AmplexRed in seedlings exposed to 200 mM NaCl for 2 hours (as described in [47], [48]. As can be seen in Figure 6, a decreased sensitivity to NaCl in the EHD1 overexpressing seedlings correlates with a decrease in ROS production in response to the exposure to NaCl, while an increase in NaCl sensitivity in the knock-down seedlings correlates with an increase in ROS production in response to NaCl treatment. Once again, the EHD1 mutant lacking the EH domain behaves like an EHD1 knock-down while the EHD1 mutant lacking the coiled-coil domain behaves similarly to EHD1 overexpressing seedlings.

**Figure 7:**
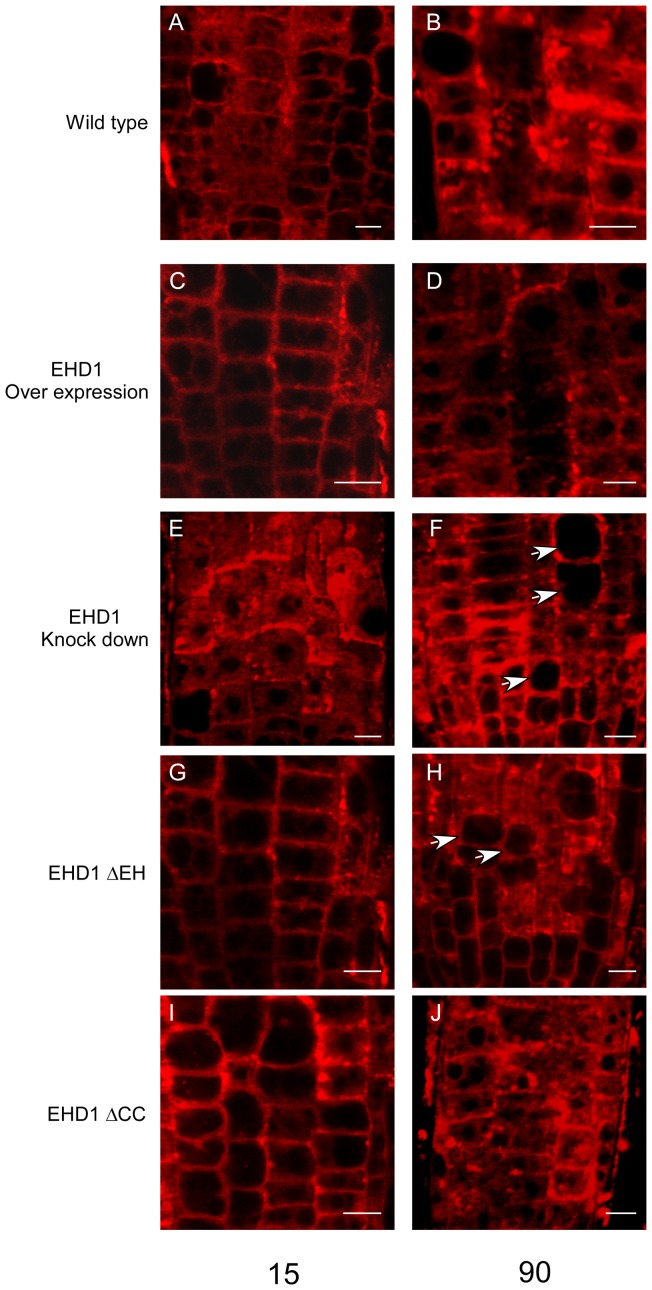
Effect of NaCl treatment on Arabidopsis seedling roots. 7–10 day old transgenic seedlings were floated on a 200 mM NaCl solution supplemented with 5 µM Fm-4-64 for different time points (as indicated) and then washed. Root sections were visualized under a laser-scanning confocal microscope. (A, B) wild type; (C, D) EHD1 overexpressing; (E, F) EHD1 knock-down; (G, H) EHD1-ΔEH overexpressing; (I, J) EHD1-ΔCC overexpressing. Scale bar  = 10 µm. Arrowheads indicate round cells that appear to have lost their osmotic integrity.

**Figure 8:**
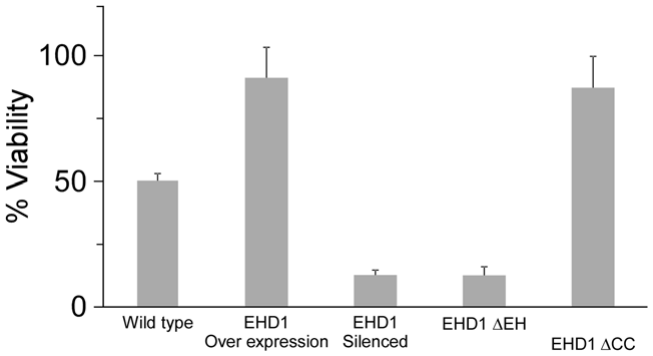
Viability of Arabidopsis seedlings treated with NaCl. Seedlings were floated on a 200 mM NaCl solution for 24 hours and then stained for viability with Neutral red. Values represent mean ± SE of 4 experiments.

To further examine the salt tolerance/sensitivity phenotype, seedlings of all types were examined microscopically following treatment with 200 mM NaCl. As can be seen in Figure 7A, wild-type seedlings floated on 200 mM NaCl for 15 minutes contain an abundance of Fm-4-64 vesicles varying in size, with the exception of the vacuole being free of such vesicles. These vesicles are fewer in number in EHD1 overexpressing cells (Figure 7C). In EHD1 knock-down cells, in addition to the salt-induced vesicles, we can see aggregation of Fm-4-64 vesicles into “clumps”, often invading the vacuolar space and creating a “smooth” appearance to the cell (Figure 7E) after 90 min of salt exposure, the *EHD1* knock-down seedlings exhibit root cells which have lost their characteristic shape and have become more rounded, probably due to the osmotic pressure (Figure 7E, F). Wild-type cells do not typically exhibit this phenotype in such a time frame. Once again, the EH domain deletion (Figure 7G, H) and the coiled-coil deletion (Figure 7I, J) behave similarly to the EHD1 knock-down and EHD1 overexpressing cells, respectively. Loss of metabolic viability of the root cells typically occurred in EHD1 knock down and EH domain deletion overexpressing earlier than in the wild-type seedlings. Figure 8 shows that after 24 hours of incubation in 200 mM NaCl, EHD1 knock down and EH domain deletion overexpressing cells have lost viability, as quantified by Neutral Red staining [49], while wild-type seedlings are still viable, and EHD1 or the coiled-coil deletion over expressing cells possess even higher viability.

Our results show that endocytosis is involved in plant salt stress coping mechanisms and indicate a role for EHD1 in salinity stress tolerance.

## Discussion

In this work we examined the function of Arabidopsis EHD1, a protein we had previously isolated and found to be endocytosis related [25]. We had previously observed that EHD1 localizes to apparently endosomal vesicles in plant cells; here we show that EHD1 is localized to RabA and RabD positive vesicles. Mammalian EHD1 was reported to co-localize with mammalian Rab11 in the endocytic recycling compartment [50]. Additionally, mutant forms of EHD1 can affect Rab11 localization and cycling [50]. Though a *per se* endocytic recycling compartment has not been reported in plants, RabA1e has been reported to be highly BFA sensitive and mark a recycling endosome in plants [37] and indeed, this class of endosomes also contain plant EHD1, strengthening the indication that this class of endosome may have recycling functions in plant cells. Further, EHD1 was found to localize to endosomes which contain plant RabD2b. This could indicate an overlap between RabA and RabD on recycling endosomes in plant cells, or perhaps indicate that EHD1 has additional functions in cellular trafficking in plants, perhaps relating to ER to golgi and/or inter golgi trafficking pathways which involve RabD [40]. Interestingly, mammalian EHD1 and EHD3, which are equally homologous to plant EHD1, were reported to be involved in retrograde transport from endosomes to golgi [35], [36]. Possibly, plant EHD1 performs transport functions attributed to both mammalian EHD1 and EHD3. At any rate, there are many Rab proteins in plant cells and possible overlap in plant Rab functionalities has been discussed at length. It is also possible that organelles in which recycling and/or sorting processes take place in plant cells can have additional functionalities. In addition to RabA/EHD1 containing endosomes, a golgi associated endosomal compartment to which EHD1 and RabD are localized in plants may mimic certain functions of the mammalian ERC.

To further characterize the functionalities of a multi-domain containing protein, we created mutant protein forms possessing a deletion of the EH domain or the coiled-coil domain, both domains being responsible for protein-protein interactions [26], [51], [52]. We found that EHD1 lacking the coiled-coil domain continues to reside on endosomal structures and co-localize with RabA and RabD proteins, while EHD1 lacking the EH domain is almost completely excluded from these vesicles. The EH domain appears to be important for the localization of EHD1, as was demonstrated in mammalian cells [32], [50]. Interestingly, an EH domain deletion mutant in mammalian EHD1 was found to lose its colocalization with Rab11, and further, to cause Rab11 to cluster in the peri-nulcear area, possibly as a result of impaired Rab11 recycling [50]. The same study found that a deletion mutant in the EH domain in mammalian EHD2 did not significantly affect the localization of the protein, and did not cause Rab11 to cluster in the peri-nulcear area, indicating that mammalian EHD2 does not affect recycling in the same manner as mammalian EHD1 [50]. Our own study relating to plant EHD2 also demonstrated that the EH domain is not crucial in its examined endocytic function [53].

We show here that EHD1 is involved in recycling, as was reported in C. elegans and mammalians [30], [50], [51], [54] and reducing its expression causes a delay in recycling. Interestingly, plants overexpressing EHD1 exhibited apparently accelerated recycling. Once again, overexpression of a deletion in the coiled-coil domain behaves similarly to overexpression of the wild-type protein, while a deletion in the EH domain behaves like a knock-down mutant and possesses delayed Fm-4-64 internalization and delayed recycling, similar to EHD1 knock-out mice [29].

Attempting to elucidate the function of EHD1 in plants, we demonstrated that overexpression of EHD1 confers salt tolerance, while seedlings knocked-down in EHD1 have increased NaCl sensitivity as compared with wild-type seedlings. Once again, the deletion in the EH domain behaves like an EHD1 knock down. The deletion in the coiled-coil domain conferred increased viability and decreased ROS production in response to salt stress as compared with wild-type seedlings (as detailed below), similarly to EHD1 overexpression, but did not confer increased germination on salt containing media, behaving instead like the wild type seeds in this instance.

We also examined the production of ROS as a stress indicator in response to NaCl treatment [48], and found that decreased sensitivity to NaCl in the EHD1 overexpressing seedlings correlates with a decrease in ROS production in response to the exposure to NaCl, while an increase in NaCl sensitivity in the knock-down seedlings correlated with an increase in ROS production in response to NaCl treatment. ROS production is a ubiquitous mechanism at play upon induction of cell damage; it seems that NaCl induced damage operates at least in part through induction of ROS. Possibly, enhancing salt tolerance causes a decrease in the induction of ROS and thus reduces the cellular damage caused by NaCl. Interestingly, the sensitivity to salt damage correlates with the endosomal localization. The mutant lacking the EH domain behaves like an EHD1 knock-down while the mutant lacking the coiled-coil domain behaves similarly to EHD1 overexpressing seedlings. This would suggest that the relative salt tolerance conferred by EHD1 may require intact localization and/or recycling function of the protein. One optional mechanism may be increased salt clearance in seedlings possessing increased recycling levels; simplistically, it is possible that proteins in charge of salt clearance are able to function more rapidly.

Vesicle trafficking seems to be involved in salt tolerance. As in the case of our EHD1 Knock-down seedlings, the Arabidopsis mutant *tno-1* displays delayed formation of BFA bodies and increased sensitivity to salt stress [55]. TNO1 is a SNARE binding protein involved in vacuolar trafficking and salt tolerance, potentially via roles in vesicle fusion and in maintaining TGN structure or identity.

We demonstrate here that plant EHD1 is an endocytic recycling protein; similar to what was reported for EHD1 in other organisms. The EH domain appears to be crucial for this function. Research into plant recycling is still in its infancy and additional advances are required before the exact pathway of recycling in which EHD1 functions can be elucidated. The involvement of EHD1 in salt tolerance may open new avenues for improving salinity tolerance by specifically modifying EHD1 expression and/or recycling mechanisms, as they become elucidated.

## Materials and Methods

### Plant and cell culture material and growth conditions

*Nicotiana benthamiana* and *Arabidopsis thaliana* cv *Columbia* were grown from seeds under greenhouse conditions.

Transgenic plants were either germinated on the appropriate sterile selective solid media and transferred to soil 2–4 weeks after germination, or, for imaging, were germinated upright in desired media containing 0.8% plant agar.

### Vectors

*AtEHD*1 was cloned in the sense orientation upstream of the *GFP* gene into the binary vector pBINPLUS between the 35S-Ω promoter containing the translation enhancer signal and the Nos terminator, generating *Pro_35S_: AtEHD1-GFP*. Primers used to clone AtEHD1 are disclosed in [25].

For silencing in Arabidopsis, a segment of *AtEHD1* (474 bp from residue 1 to residue 474) was cloned in the pKANNIBAL vector in both the sense and the anti-sense orientation, flanking the Pdk intron [56]. The construct was sub-cloned into the binary vector pART27 [57] and used for transforming *Arabidopsis* plants.

The truncation mutants were generated by amplifying fragments of the cDNA as desired, with the following primers: EHD1_ΔEH FOR: 5′atgcttattagcgatgttg (used with the EHD1 reverse primer); EHD1 ΔCC(1) REV: CATTATCGCTGGCATCTCC (used with the EHD1 forward primer to generate the first fragment); EHD1-ΔCC(2) FOR: TTTGGAAAGGTACAAAGAG (used with the EHD1 reverse primer to generate the second fragment; the fragments were then ligated to form EHD1 ΔCC); In addition to the forward and reverse primers disclosed in [25].

All constructs were cloned in pBINPLUS as described above for AtEHD1. The constructs were electroporated into *Agrobacterium tumefaciens* GV3101 and the bacteria used for transient expression assays.

The Wave lines constructs were obtained from Prof. Geldner [37].

### Stable and transient transformation

Arabidopsis plants were transformed as previously described [58].

Transient expression was performed as previously described [59]. Briefly, the *AtEHD* constructs were cloned in pBINplus [60] and introduced by electroporation into *Agrobacterium tumefaciens* strain GV3101. *Agrobacterium* were grown in LB medium overnight, diluted into an induction medium (50 mM MES pH-5.6, 0.5% (w/v) glucose, 1.7 mM NaH_2_PO_4_, 20 mM NH_4_Cl, 1.2 mM MgSO_4_, 2 mM KCl, 17 µM FeSO_4_, 70 µM CaCl_2_ and 200 µM acetosyringone) and grown for an additional 6 h until OD_600_ reached 0.4–0.5. The *Agrobacterium* culture was diluted to OD_600_ = 0.05–0.2, and the suspensions were injected with a needleless syringe into the leaves of 7–8 week old tobacco plants. Leaves were observed for protein expression 24 to 72 h after injection.

### Confocal microscopy

Cells were analyzed using a Zeiss LSM-510-Meta confocal laser scanning microscope (Zeiss, Oberkochen, Germany) with the following configuration: 30 mW Argon and HeNe lasers, 458, 477, 488, 514 and 568 maximum lines. All images depict single sections, except where indicated otherwise. Contrast and intensity for each image were manipulated uniformly using Adobe Photoshop and/or ImageJ software.

### BFA, NaCl, Fm-4-64 and Neutral Red treatments/staining

Roots of 1–2 week old Arabidopsis seedlings were floated on a solution of Brefeldin A (50 µM, Sigma) or 200 mN NaCl or water, containing 5 uM Fm-4-64 for desired time points. Fm-4-64 staining was examined under a confocal laser scanning microscope. For viability, Roots were stained with 4 µM Neutral Red in 0.2 MS as described in [49]. For germination experiments, seeds were germinated on 0.5 MS alone or supplemented with 200 mM NaCl. For germination statistics the criterion used was radical emergence.

### Semi-Quantitative RT-PCR

Total RNA was extracted from 7 day old *Arabidopsis thaliana seedlings* (wild type and transgenic) using the SV Total RNA Isolation System (Promega, Madison, WI) according to manufacturer's instructions. 4 µg of RNA were converted to cDNA using M-MLV reverse transcriptase (Promega, Madison, WI). One µl of each reverse transcriptase reaction was used as a template in a PCR reaction containing the following specific primer pairs: Cyclophilin (at2g36130) AGTCCGCCGGAGGTTACGCT (as normalizer) and TGGATCGGCCTGTCGGTGTT and for EHD1 GGGGATCCATGGAGATCGAATCCGTCGC and CTGCTTGAACTGCTACTGTG. To monitor the expression of EHD1 forms in the transgenic plants a 4ul aliquot of each reverse transcriptase reaction was used as template in a PCR reaction containing the following primers EHD1-ΔCC(2) FOR (TTTGGAAAGGTACAAAGAG) and GFP REV (GGGCCAGGGCACGGGCAGCTT). The amplified fragment was 370 bp long.

Quantification of the resultant PCR reactions was performed using ImageJ software.

### ROS quantification

ROS were quantified as described in [47]. Roots of 1 week old Arabiopsis seedlings were floated on a 200 mM NaCl solution for 2 hours, then washed and stained with Amplex® Red (Invitrogen). ROS was quantified by measuring pixel intensity of pictures taken with a Zeiss fluorescent microscope.

## Supporting Information

Figure S1 **Co-localization of EHD1 with Fm-4-64 following BFA treatment.** 7–10 day old transgenic seedlings were floated on a 50 µM BFA solution supplemented with 5 µM Fm-4-64 for 30 minutes and then washed. Root sections were visualized under a laser-scanning confocal microscope. Scale bar  = 10 µm.
(TIF)

Figure S2 **The effect of BFA and salt treatment on EHD1 and EHD2 knock-down seedlings.** 7–10 day old transgenic seedlings were floated on a 200 mM NaCl solution for 60 minutes or a 50 µM BFA solution for 30 minutes, both supplemented with 5 µM Fm-4-64, and then washed. Root sections were visualized under a laser-scanning confocal microscope. Scale bar  = 10 µm.
(TIF)

Figure S3 **Expression of EHD1 forms in transgenic Arabidopsis plants.** cDNA was prepared from 5–6 day old transgenic seedlings as indicated. The presence of the GFP tagged EHD1/ΔEH/ΔCC cDNA was confirmed by PCR.
(TIF)
