# Supplementary figures and images for: EHD1 Functions in Endosomal Recycling and Confers Salt Tolerance
Source: PLoS One. 2013 Jan 14;8(1):e54533. doi: 10.1371/journal.pone.0054533 (PMC3544766; doi:10.1371/journal.pone.0054533)

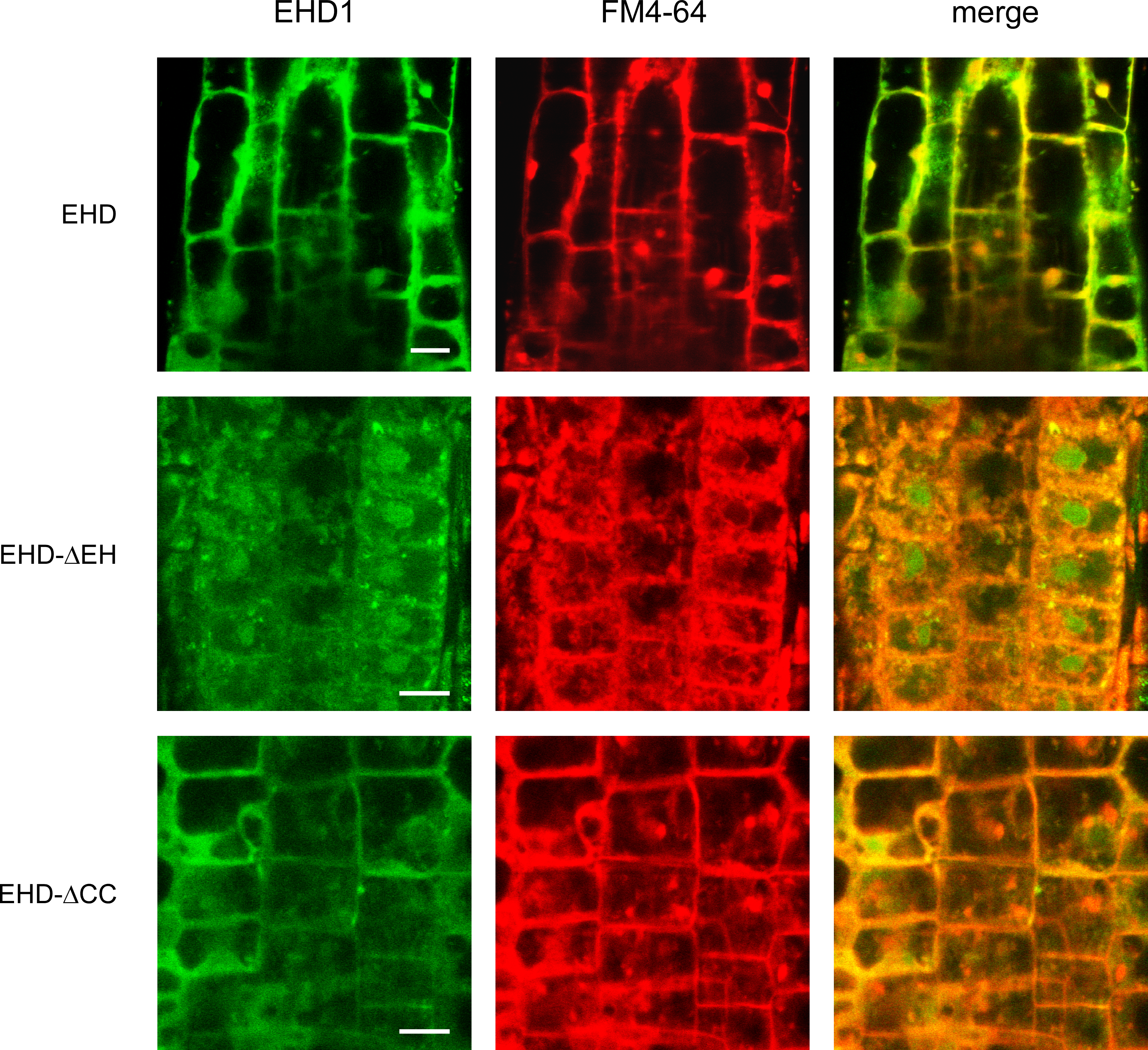

Supplement: Figure S1 — Co-localization of EHD1 with Fm-4-64 following BFA treatment. 7–10 day old transgenic seedlings were floated on a 50 µM BFA solution supplemented with 5 µM Fm-4-64 for 30 minutes and then washed. Root sections were visualized under a laser-scanning confocal microscope. Scale bar = 10 µm. (TIF) [file pone.0054533.s001.tif]

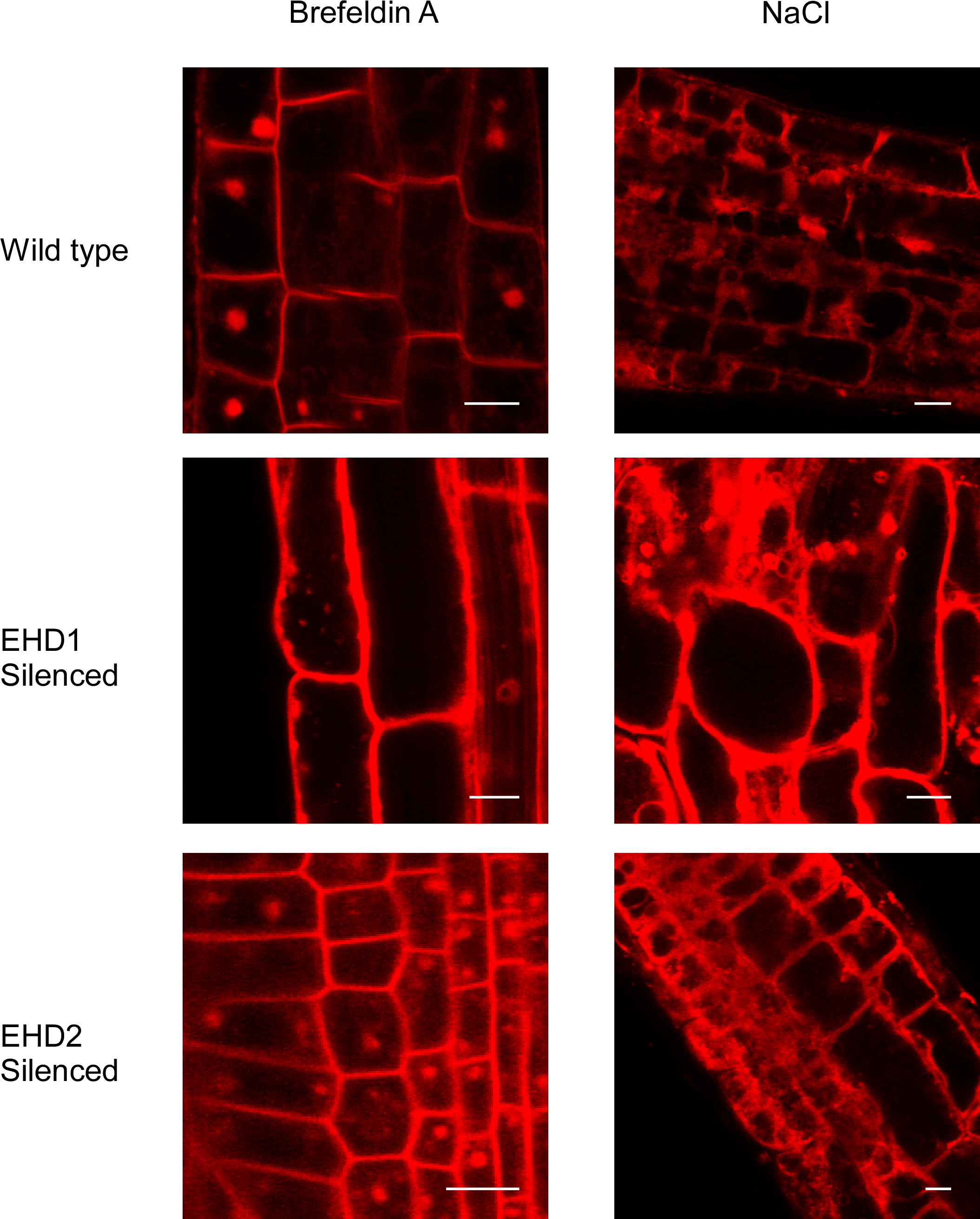

Supplement: Figure S2 — The effect of BFA and salt treatment on EHD1 and EHD2 knock-down seedlings. 7–10 day old transgenic seedlings were floated on a 200 mM NaCl solution for 60 minutes or a 50 µM BFA solution for 30 minutes, both supplemented with 5 µM Fm-4-64, and then washed. Root sections were visualized under a laser-scanning confocal microscope. Scale bar = 10 µm. (TIF) [file pone.0054533.s002.tif]

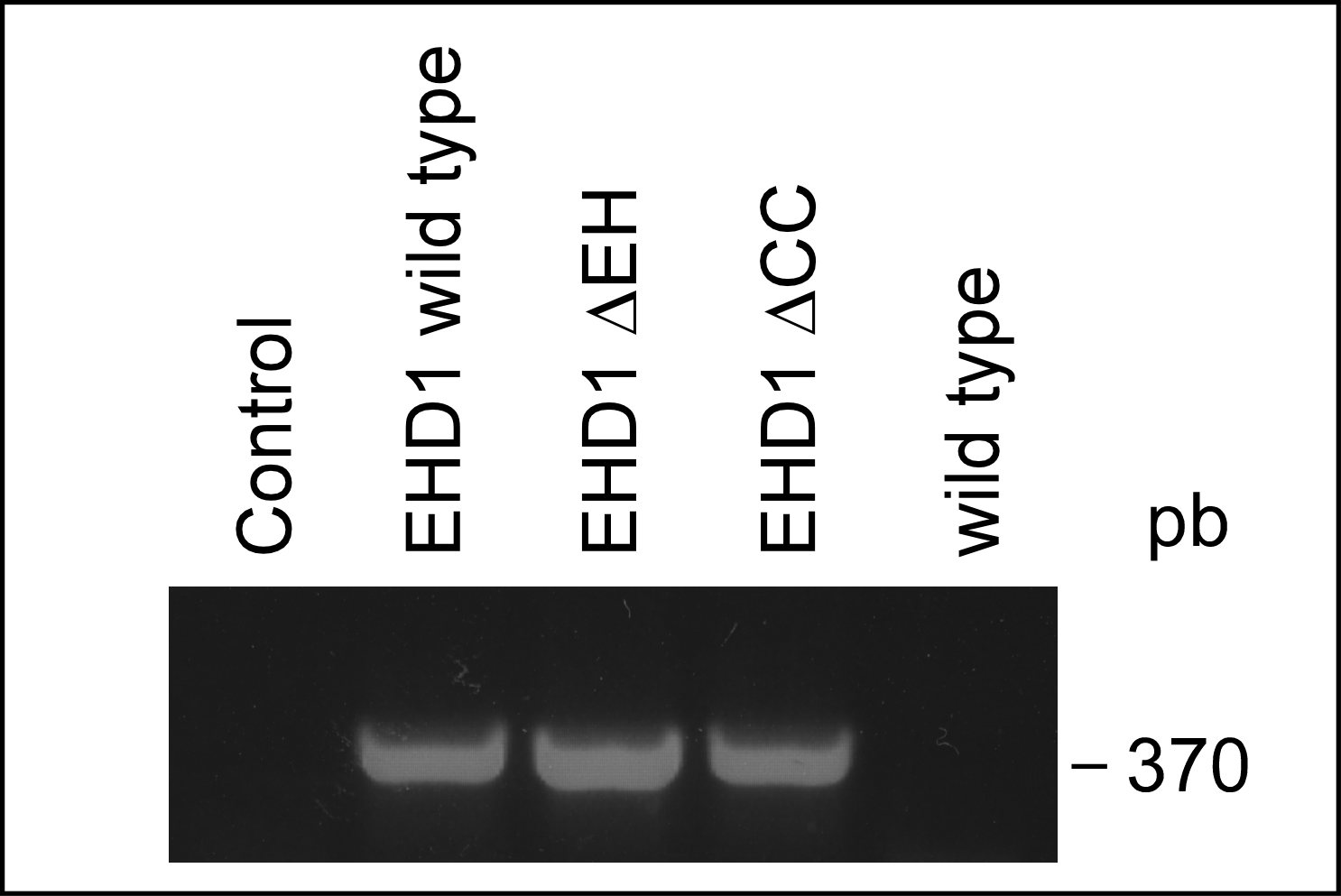

Supplement: Figure S3 — Expression of EHD1 forms in transgenic Arabidopsis plants. cDNA was prepared from 5–6 day old transgenic seedlings as indicated. The presence of the GFP tagged EHD1/ΔEH/ΔCC cDNA was confirmed by PCR. (TIF) [file pone.0054533.s003.tif]
